# Subjective and objective cognition 6-week post-coronary artery bypass graft surgery: A descriptive pilot study

**DOI:** 10.4102/sajpsychiatry.v26i0.1470

**Published:** 2020-07-09

**Authors:** Ntokozo N. Ngcobo, Andrew Tomita, Suvira Ramlall

**Affiliations:** 1 Department of Psychiatry, Nelson R. Mandela School of Medicine, University of KwaZulu-Natal, Durban, South Africa; 2 Discipline of Psychiatry, School of Clinical Medicine, University of KwaZulu-Natal, Durban, South Africa

**Keywords:** coronary artery disease, coronary artery bypass graft surgery, cognitive screening, subjective memory impairment, cognitive decline, depression

## Abstract

**Background:**

Coronary artery bypass graft (CABG) surgery has been found to be associated with post-operative cognitive decline. Despite the large and growing numbers being conducted in South Africa, the associated or ensuing cognitive symptoms or impairment have received little research attention.

**Aim:**

The aim of this pilot study was to describe the nature and extent of subjective cognitive complaints (SCCs) and objective cognitive impairments in patients 6-week post-CABG surgery in a clinical sample in KwaZulu-Natal (KZN) Province, South Africa.

**Setting:**

A cross-sectional survey was conducted among outpatients attending their 6-week post-CABG surgical review at a cardiology clinic in a KZN provincial hospital.

**Method:**

Socio-demographic and clinical data were captured, with SCCs being determined by using standardised questions; cognition was assessed with the Montreal cognitive assessment (MoCA).

**Results:**

The mean age of the sample (*n* = 28) was 58.72 years. The mean MoCA score was 23.96 (SD = 4.32); 60.71% (*n* = 17) screening positive (< 25/30) and more likely to be older, male, hypertensive and diabetic. A third (*n* = 9; 35.71%) reported at least one new SCC; their mean age was 55.36 years which was lower than those without subjective complaints (59.81).

**Conclusions:**

Subjective and objective cognitive impairments were evident in patients 6-week post-CABG surgery identifying a need for longitudinal cognitive screening both pre- and post- operatively in patients undergoing CABG surgery.

## Introduction

It has been estimated that coronary artery disease (CAD) will be the single largest cause of the global disease burden worldwide by the year 2030.^1^ The increasing prevalence of coronary heart disease (CHD) has also led to an increased need for surgical interventions, with coronary artery bypass graft (CABG) surgery being among the most commonly performed major operations.^2^ However, the neurological sequel with the highest incidence post-cardiac surgery post-operative cognitive decline (POCD).^3^

Of the patients who undergo revascularisation surgery, 1.4% – 3.8% experience vascular events. The risk factors include atherosclerotic disease and increased age, with surgery and anaesthetic-related factors also being implicated.^3,4^ Coronary artery bypass graft surgery is associated with significant cerebral morbidity, ranging from a stroke, which is well documented, to subtle changes in cognition or neurobehavioural problems, which often occur much later and are therefore missed and thus poorly documented.^5^ The incidence of adverse cerebral events after coronary artery bypass surgery is 6.1%, of which half are type I (focal injury, coma or stupor at discharge) and half are type II (deterioration in intellectual function, memory defect or seizures), the incidence of both types is increasing with age.^6^

Post-operative cognitive decline is defined as a *new* cognitive impairment that arises after a surgical procedure, its diagnosis requiring both pre- and post-operative psychometric testing, with symptoms ranging from memory impairment to diminished performance on a range of intellectual tasks.^3,7,8,9^ Post-operative cognitive impairment is traditionally divided into early and late types, the former dysfunction manifesting in the first few days or weeks after surgery, being characterised by memory difficulties and decreased visual attention.^3,5^ The prevalence of POCD is higher in the early post-operative period (3–6 weeks) and decreases during the following year.^10^

International studies on cognition which are carried out in the first few days or weeks after CABG surgery have revealed a 33% – 83% prevalence of a wide range of short-term cognitive deficits, the most common being memory and visuospatial impairments.^8^ A systematic review of studies of neurocognitive dysfunction following CABG surgery found that 22.5% of patients present with a cognitive deficit 2 months after the operation, with memory being the domain with the highest frequency of decline in most studies.^11^ Monk et al. reported a POCD incidence of 36.6% in patients discharged post-surgery and aged 18–39 years, 30.4% for those aged 40–59 years and 41.4% for patients aged 60 years and above.^7^

In a South African study including 19 patients who were assessed 1-day pre- and 1-week post-operatively, 63% demonstrated cognitive change in various neuro-psychological domains, with significant post-operative decline noted for specific tasks of the Grooved Pegboard Test (fine motor co-ordination), the Rey Auditory Verbal Learning Test (verbal learning and memory) and the Trail Making Test (motor speed and attention).^12^ The reasons for the widely varying incidence of cognitive decline are multifactorial, and make comparisons difficult; firstly, there are a large number of neuro-psychological tests that assess the different cognitive domains.^13,14^ Secondly, the intervals between cognitive screening pre-operatively and the operation date vary across studies, and range from weeks to months.^14^ Finally, the wide variation in the definition of POCD used across studies accounts for differing incidence and prevalence rates.

With increasing longevity and improving healthcare, there also needs to be a focus on the quality of life of patients surviving cardiac disease. While approximately 8400 coronary bypass operations are performed annually in South Africa,^15^ the presence, nature and severity of associated or ensuing cognitive symptoms or impairment have received little research attention, especially in low- and middle-income countries. The aim of this pilot study was to describe the nature and extent of subjective cognitive complaints (SCCs) and objective cognitive impairment in patients 6-week post-CABG surgery in a clinical sample in KwaZulu-Natal (KZN) Province, South Africa.

## Methodology

### Study setting and population

A cross-sectional questionnaire survey of post-CABG surgery patients attending the cardiology clinic at a central specialised provincial hospital in Durban, KZN, at which all such public sector procedures are performed, was conducted in 2018. Eligible participants included patients attending their first post-surgery review 6 weeks after surgery, had a minimum of 8 years of formal schooling and were able to speak, read and write in English to enable the cognitive screening tool to be administered. Those with an existing diagnosis of a cognitive disorder, or who reported cognitive symptoms ante-dating the surgery, were excluded.

Convenience sampling was used, with every consecutive patient being screened for eligibility. The 6-week post-operative visit was specifically chosen to exclude reversible peri-operative surgical and anaesthetic factors that may temporarily impact on cognitive functioning. Assessments after 6 weeks would be confounded by the effects of the normal progression of age-related cognitive decline.

### Study measures and instruments

Data collection was conducted by making use of a socio-demographic questionnaire, clinical information pertaining to the surgical procedure and anaesthesia, a subjective cognitive assessment, the Montreal cognitive assessment (MoCA) test which is an objective measure of cognitive functions and the cardiac depression scale (CDS).

SCC screening questions were based on questions derived by the authors based on clinical experience. In face validity was confirmed by psychologists and psychiatrists working with patients with cognitive impairment.

The MoCA is a cognitive screening test that assesses a broad range of cognitive domains that are scored out of 30 points, a score of ≤ 25 being classified as a screen positive. Post-operative cognitive decline, as described by the International Society of Postoperative Cognitive Dysfunction, is defined as post-operative deficits observed in a patient in one or more discrete areas used to describe the mental state, such as memory, attention, concentration, psychomotor speed, visuospatial ability and executive function.^16^ The MoCA was chosen as it assesses for these domains as well as for naming, language, abstraction and orientation.^17^ The gold standard for assessing cognitive functioning includes clinical assessment and neuro-psychological testing.

The busy study site is the only one in KZN that caters to the public sector, which precluded pre-operative assessments, as patients are admitted a day prior to the scheduled operation, while the post-operative assessments had to be completed within the constraints of a single follow-up visit because of the financial implications for participants who are referred from outlying hospitals.

The CDS is the only instrument designed to measure depression in cardiac patients. The tool was designed to exclude somatic symptoms in its screening and has good sensitivity to measure moderate to severe depression in the presence of cardiac disease.^18^ The scale consists of 26 items scored on a Likert scale (1–7);, the higher the score, the worse the depressive symptomology. For the purposes of this study, a cut-off score of 89 was used, with a score of 90 and above indicating depression.^18^

### Statistical analysis

All statistical analyses were performed using STATA 15. Firstly, the socio-demographic, clinical (e.g. hypertension, diabetes, substance use, types of bypass surgery) and cognitive characteristics (subjective and objective) of participants were summarised using median and interquartile range (IQR) for continuous variables, and as proportions (%) for categorical variables. Secondly, we summarised socio-demographic and clinical characteristics of participants by cognitive characteristics (subjective and objective).

### Ethical considerations

The study was approved by the Biomedical Research Ethics committee at the University of KwaZulu-Natal and the KwaZulu-Natal Department of Health. All research participants provided written informed consent.

## Results

### Socio-demographic and clinical profile

The sample consisted of 28 adults, mainly males, and with a median age of 58.72 years (IQR = 11.53), while 85.71% (*n* = 24) had on-pump procedures (Table 1). The majority were Indian (67.86%), and all had a high school education, with one also having a tertiary qualification. The main source of income for 35.71% was from state pension funds, while the majority were unemployed (57.14%). More than half (57.14%) were married, with most living in urban areas (92.86%), while 60.71% were on treatment for hypertension and 46.43% for diabetes. Participants with a prior history of mental illness or neurologic deficit were excluded from the study, with no new neurologic events being reported by the participants. Half were smokers and five (17.86%) reported alcohol use, while two had a family history of psychiatric illness.

**TABLE 1:** Socio-demographic and clinical profile of study participants (*N* = 28).

Socio-demographics	Variables	*N*	%
Age category	Less than 50	4	14.29
	50–59	13	46.43
	60 and older	11	39.23
Gender	Female	7	25.00
	Male	21	75.00
Race	Black people	5	17.86
	Mixed race people	1	3.57
	Indian people	19	67.86
	White people	3	10.71
Highest level of educational	High school	27	96.43
	Tertiary	1	3.57
Employment status	Employed	2	7.14
	Pensioner	10	35.71
	Unemployed	16	57.14
Marital status	Divorced or widow	7	25.00
	Married	16	57.14
	Single	5	17.86
Typology	Rural	2	7.14
	Urban	26	92.86
Hypertension	No	11	32.39
	Yes	17	60.71
Diabetes	No	15	53.57
	Yes	13	46.43
Tobacco	No	14	50.00
	Yes	14	50.00
Alcohol	No	23	82.14
	Yes	5	17.86
Family history of mental illness	No	26	92.86
	Yes	2	7.14
Bypass graft surgery	Off-pump	4	14.29
	On-pump	24	85.71
Duration of CABG surgery	114 min	0	0.00
	≥ 114 min	28	100.00

CABG, coronary artery bypass graft.

### Subjective and objective cognitive status post-surgery

Overall, one-third of study participants (*n* = 9; 32.14) reported at least one SCC.

The mean MoCA (i.e. objective cognitive measure) was 23.96 (standard deviation [SD] = 4.32) and median 24 (IQR was 7).

Approximately two-thirds (*n* = 17; 60.71%) screened positive on the MoCA. The majority (*N* = 21; 75%) reported no subjective change and less than a quarter (*n* = 6; 21.43%) indicated a worsening in their short-term memory following surgery. The majority (89.29%) reported no change and 7.14% (*N* = 2) reported a worsening of their long-term memory. With respect to speed of thinking, 10.71% reported a decrease while 89.29% reported no change. A minority (10.71%) reported a decline in community engagement post-surgery. A subjective improvement in short- and long-term memory was experienced by one participant each (Table 2).

**TABLE 2:** Subjective and objective cognition status post-surgery (*N* = 28).

Variable	Status	*N*	%
Short-term memory (subjective)	Improved	1	3.57
	Same	21	75.00
	Worse	6	21.43
Long-term memory (subjective)	Improved	1	3.57
	Same	25	89.29
	Worse	2	7.14
Thinking speed (subjective)	Improved	0	0.00
	Same	25	89.29
	Worse	3	10.71
Task completion time (subjective)	Decreased	1	3.57
	Same	17	60.71
	Increased	10	35.71
	Improved	0	0.00
Community participation (subjective)	Same	25	89.29
	Worse	3	10.71
At least one complaint (subjective)	No	19	67.86
	Yes	9	32.14
MoCA score (objective)	-	24†	7‡
MoCA category (objective)	MoCA –	11	39.29
	MoCA +	17	60.71

MoCA, Montreal cognitive assessment;

† , median;

‡ , interquartile range.

MoCA + is based on score less than or equal to 25.

### Socio-demographic and clinical profile distribution by subjective and objective cognition status

Table 3 illustrates the socio-demographic and clinical profile distribution by subjective and objective cognition status. The median age of the 17 participants who screened positive on the MoCA was 59.68 (IQR = 6.60) compared to 52.67 years (IQR = 9.21) in the 11 who screened negative. All participants had a minimum of high school level of education, with the person having a tertiary education being 44 years old, unemployed and screening negative on the MoCA; both the employed participants also screened negative. Of the 10 pensioners (65+ years), eight screened positive. Those who were married (68.8%) and divorced or widowed (71.4%) were more likely to screen positive than those who were single (20%). Of those who screened positive, 11/17 (61%) were married, 61% were hypertensive and 47% were diabetic. Fifty per cent of the participants who had off-pump surgeries screened positive versus 62.5% of those who had on-pump operations. Participants reporting subjective cognitive decline (SCD) were, on average, younger than those with none (55.36 vs. 59.68 years); SCD was more common in participants who were male, unemployed, married, hypertensive and those who had on pump procedures and less frequent among participants reporting alcohol use (1/9).

**TABLE 3:** Socio-demographic and clinical profile distribution by subjective and objective cognition status (*N* = 28).

Socio-demographics	Variables	At least one complaint (No)		At least one complaint (Yes)		MoCA screen −		MoCA screen +	
		*n* = 19	%	*n* = 9	%	*n* = 11	%	*n* = 17	%
Gender	Female	5	71.43	2	28.57	2	28.57	5	71.43
	Male	14	66.67	7	33.33	9	42.86	12	57.14
Race	Black poeple	4	80.00	1	20.00	1	20.00	4	80.00
	Mixed race poeple	1	100.00	0	0.00	1	100.00	0	0.00
	Indian people	13	68.42	6	31.58	8	42.11	11	57.89
	White people	1	33.33	2	66.67	1	33.33	2	66.67
Educational attainment (highest)	High school	18	66.67	9	33.33	10	37.04	17	62.96
	Tertiary	1	100.00	0	0.00	1	100.00	0	0.00
Employment status	Employed	1	50.00	1	50.00	2	100.00	0	0.00
	Pensioner	9	90.00	1	10.00	2	20.00	8	80.00
	Unemployed	9	56.25	7	43.75	7	43.75	9	56.25
Marital status	Divorced or widow	4	57.14	3	42.86	2	28.57	5	71.43
	Married	11	68.75	5	31.25	5	31.25	11	68.75
	Single	4	80.00	1	20.00	4	80.00	1	20.00
Hypertension	No	10	90.91	1	9.09	5	45.45	6	54.55
	Yes	9	52.94	8	47.06	6	35.29	11	64.71
Diabetes	No	11	73.33	4	26.67	7	46.67	8	53.33
	Yes	8	61.54	5	38.46	4	30.77	9	69.23
Tobacco	No	10	71.43	4	28.57	3	21.43	11	78.57
	Yes	9	64.29	5	35.71	8	57.14	6	42.86
Alcohol	No	15	65.22	8	34.78	9	39.13	14	60.87
	Yes	4	80.00	1	20.00	2	40.00	3	60.00
Bypass graft surgery	Off-pump	2	50.00	2	50.00	2	50.00	2	50.00
	On-pump	17	70.83	7	29.17	9	37.50	15	62.50

Note: Age (mean years) for at least one complaint (No), Median = 59.81 and IQR = 10.62. For at least one complaint (Yes), Median = 55.36 and IQR = 6.51. For MoCA screen −, Median = 52.67 and IQR = 9.21. For MoCA screen + Median = 59.68 and IQR = 6.60.

MoCA, Montreal cognitive assessment; IQR, interquartile range.

### Socio-demographic and clinical profile distribution by subjective and objective cognition risk status

The results pertaining to socio-demographic and clinical profile distribution of individuals with at least one domain of SCD (*n* = 9) and MoCA screen positive (*n* = 17) are provided in Table 4. Out of those who screened positive on the MoCA, 4/17 (23.53%) reported at least one subjective complaint, while, out of those with at least one subjective cognitive impairment (SCI), 4/9 (44.44%) screened positive on the MoCA; 4/28 (14.29%) screened positive on both subjective and objective measures of impairment; of these 3/4 (75.00%) were male, 0/4 (0.00%) consumed alcohol, 4/4 (100.00%) had the on-pump procedures, 4/4 (100.00%) were hypertensive and 2/4 (50.00%) were diabetic.

**TABLE 4:** Socio-demographic and clinical profile distribution by subjective and objective cognition risk status.

Socio-demographic	Variables	At least one subjective complaint (*n* = 9)		MoCA screen + (*n* = 17)	
		*N*	%	*N*	%
Gender	Female	2	22.22	5	29.41
	Male	7	77.78	12	70.59
Race	Black people	1	11.11	4	23.53
	Indian people	6	66.67	11	64.71
	White people	2	22.22	2	11.76
Educational attainment (highest)	High school	9	100.00	17	100.00
Employment status	Employed	1	11.11	0	0.00
	Pensioner	1	11.11	8	47.06
	Unemployed	7	77.78	9	52.94
Marital status	Divorced or widow	3	33.33	5	29.41
	Married	5	55.56	11	64.71
	Single	1	11.11	1	5.88
Hypertension	No	1	11.11	6	35.29
	Yes	8	88.89	11	64.71
Diabetes	No	4	44.44	8	47.06
	Yes	5	55.56	9	52.94
Tobacco	No	4	44.44	11	64.71
	Yes	5	55.56	6	35.29
Alcohol	No	8	88.89	14	82.35
	Yes	1	11.11	3	17.65
Bypass graft surgery	Off-pump	2	22.22	2	11.76
	On-pump	7	77.78	15	88.24

Note: Age (mean years) for at least one subjective complaint, Median = 55.36 and IQR = 6.51. For MoCA screen + Median = 59.68 and IQR = 6.60.

MoCA, Montreal cognitive assessment; IQR, interquartile range.

### Depression

One participant, who was an unemployed, married, female and diabetic, screened positive for depression, with a CDS score of 117. The participant had a MoCA score of 21 and had worsened subjective memory and thinking speed post the CABG surgery.

## Discussion

To our knowledge, this is the first study to be conducted in KZN which describes the patients subjective and objective cognitive status 6-week post-CABG surgery. Our study population showed that 21.43% reported at least one new SCD since their CABG procedure, while 60.71% screened positive on the MoCA test. Although our sample size was small, the emerging associated socio-demographic factors and profile of the 6-week post-CABG surgery subjective and objective cognitive deficits are noteworthy in several respects.

The subjective experience of improvements post-operatively in the domains of cognitive functioning that were assessed was found in only one participant. This suggests that surgery may have little impact on existing deficits nor be more relevant in preventing further deterioration.

Although there is lack of universal agreement on the validity of the SCI, our data suggest that almost a quarter (23.53%) of those who screen positive on objective cognitive assessment also experience subjective impairment and of those with subjective impairment, more than a third (44.44%) show evidence of objective impairment on screening. More than one in 10 participants screened (14.28%) had both subjective and objective impairment. Males who were hypertensive and did not consume alcohol appear to characterise those with both measures of impairment. These data, if replicated in bigger samples, have relevance for cognitive screening of a high-risk clinical population. Subjective cognitive impairment is central to the diagnosis of mild cognitive impairment (MCI),^19^ which formed the basis of the Diagnostic and Statistical Manual of Mental Disorders (DSM) 5 category of mild neurocognitive disorder (mNCD). Participants screening positive on objective tests were, on average, 4.45 years older than those screening negative; conversely, screen positives on SCI were, on average, younger than screen negatives. Subjective cognitive complaints are common in the elderly and clinically useful; however, their aetiology can be heterogeneous, and its construct is not yet adequately refined to reliably establish its association with objective impairment and relationship to progression to dementia (major neurocognitive disorder).^20,21^ Subjective awareness in younger individuals than those with objective impairment may suggest that they represent early signs of risk; conversely their lower frequency in older individuals may represent anosognosia. Nonetheless, they have potential for easy first-stage screening of individuals at risk of objective cognitive impairment, as argued for by Ramlall et al.^22^ This could make screening feasible in low resource settings where there is a dearth of mental health practitioners, and assist with detecting at-risk individuals at an earlier age.

A majority (60%) of the participants screened positive on the MoCA test. Early neuro-cognitive changes are most likely because of a combination of factors that include microemboli, relative hypotension, general anaesthesia and the overall inflammatory condition initiated by cardiopulmonary bypass (CPB) surgery.^7^

Studies have shown that neuro-cognitive deficits occur when patients are tested early after their operation, and that by 3 months, the majority have resolved, such that the cardiac surgical groups are the same as the control groups. Studies have shown that late cognitive decline after cardiac surgery is likely because of patient age and the degree of cardiovascular and cerebral vascular disease risk factors, and not because of the use of CPB.^7^ It was previously contended that patients who underwent cardiac surgery were at risk of developing dementia or Alzheimer’s disease, with recent evidence suggesting that CABG surgery was not a major risk factor for either.^7^ As a pre-operative assessment of cognition was not undertaken in our study, it cannot be assumed that the identified deficits emerged post-operatively. Nonetheless, the finding of cognitive deficits in 3/5 patients highlights a high frequency of impairment that warrants routine pre- and post-operative screening and monitoring, with preventative strategies needing to be considered, as both mild and major neuro-cognitive deficits have both cost and care implications.

The risk factors for POCD can be classified according to patient, surgical and anaesthetic factors. The mean age of screen positives was higher than the negatives (59.54 vs. 54.76 years), which is consistent with the known association between increasing age being an important risk factor for POCD.^3^ Borowicz et al.^23^ reported that the patient population undergoing CABG surgery is becoming increasingly older.

Increased age is also linked to risk factors for cardiovascular disease, namely, diabetes, atherosclerosis and hypercholesterolaemia, which contribute to POCD. Hypertension was experienced for 61% of our sample and almost half (46%) were diabetic. These factors lead to the amplification of systemic and neuronal inflammation, which precipitates widespread neuronal dysfunction, thereby contributing to POCD.^3^ Initial low pre-operative baseline scores on neuro-psychological testing indicated mild cognitive decline, which is associated with hypertension, age and low education level, and may also increase the risk of POCD.^3^ The protective effects of education on cognition are also well established in the literature; however, as nearly all the participants had a high school education, this was difficult to demonstrate in our study – the one with a tertiary education screening negative on the MoCA.^9^

Studies have suggested that shorter durations of surgery, among other factors, may reduce the incidence of POCD.^10^ While all participants underwent surgery for more than 114 min in our study, the absence of pre-operative baseline cognitive data makes it difficult to surmise any impact of the longer duration of operations on cognition. Recent studies show that, rather than the type of operation, that is, on-pump versus off-pump procedure, the degree of aortic manipulation may be the predominant cause of neurological injury after cardiac surgery, as embolic material can be derived from CPB and aortic lesions.^7^ Increased perfusion pressure equivalent to physiological conditions, hypothermia and non-extreme haemodilution are some of the other factors that may reduce the incidence of POCD.^3^ The finer details of the surgery were not included in this pilot study, but will be useful in a larger scale longitudinal study.

It has also been postulated that anaesthesia may be a possible cause of POCD, with studies comparing propofol with sevoflurane and desflurane, which are both inhalants, finding a higher incidence of cognitive dysfunction in patients after propofol-based anaesthesia.^24^ Data from our study revealed that of the 28 participants studied, 27 received propofol and all 28 received sevoflurane, which could be considered as a possible contributing factor to the high rate of positive cognitive impairment screening. It is possible that hypnotic or anaesthetic agents could worsen the insults associated with cardiac injury and cause permanent structural changes in the brain or long-term cognitive deficits, which is evidenced by the fact that impaired memory can persist for a considerable time after recovery from anaesthesia.^24^

### Limitations

This research has several limitations, the first being that the study was conducted in only one public tertiary hospital and the findings may not be generalisable to other centres in the country or to the private health sector.

Participation was voluntary, which may have resulted in the sampling being biased. Cognitive status was based on the performance on a screening tool and not on the gold standard of neuro-psychological testing. The possible contributory effect of surgery per se on cognitive status would more reliably be based on a comparison between pre- and post-operative cognitive measures.

## Conclusion

Despite the limitations, the finding that 60% of participants screened positive for cognitive impairment highlights a need for cognitive screening both pre- and post-operatively in patients undergoing CABG surgery. Patients with cognitive impairment are less likely to adhere to their treatment, thus worsening the clinical condition. Early identification, through screening, will allow for referral for more extensive neuro-psychological assessment, which will assist with early diagnosis, risk reduction strategies, holistic treatment, rehabilitation for affected patients and support for caregivers. Use of culturally adapted and locally validated cognitive screening tools must be considered for future studies, which should extend longitudinally from pre-operatively to at least 6-month post-CABG surgery to accommodate the confounding temporary effects of intra- and post-operative factors.
